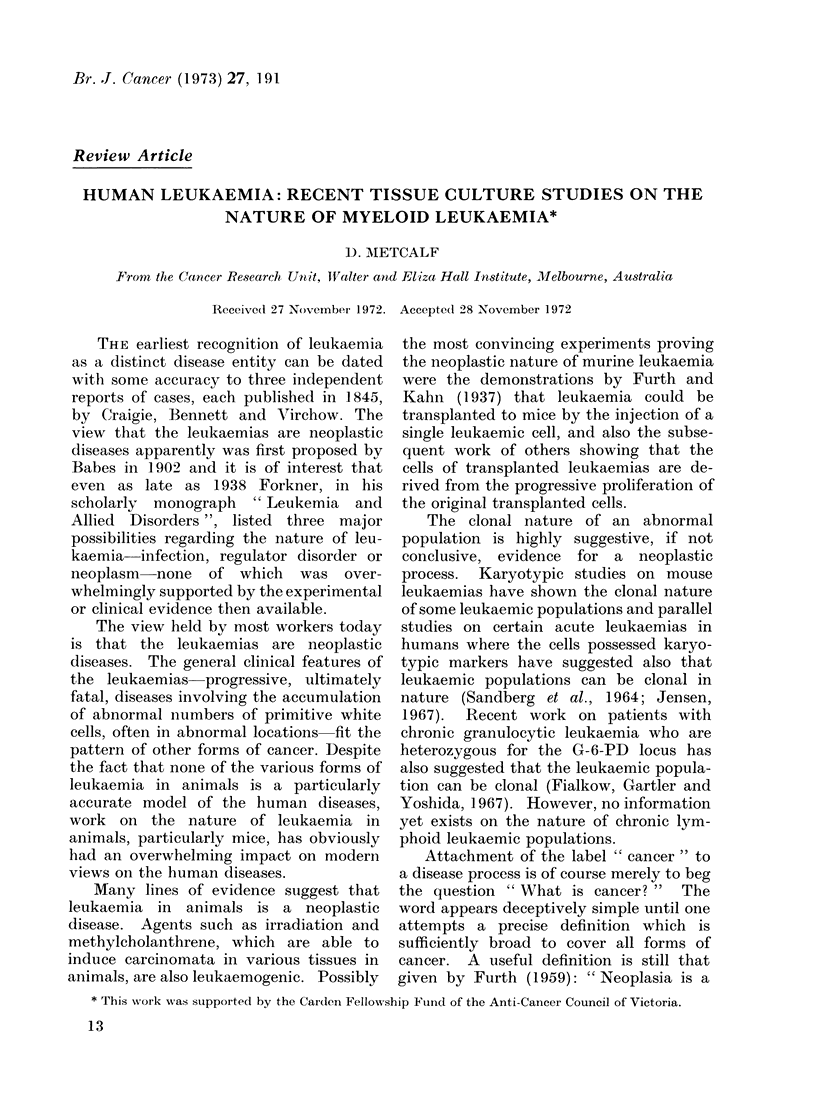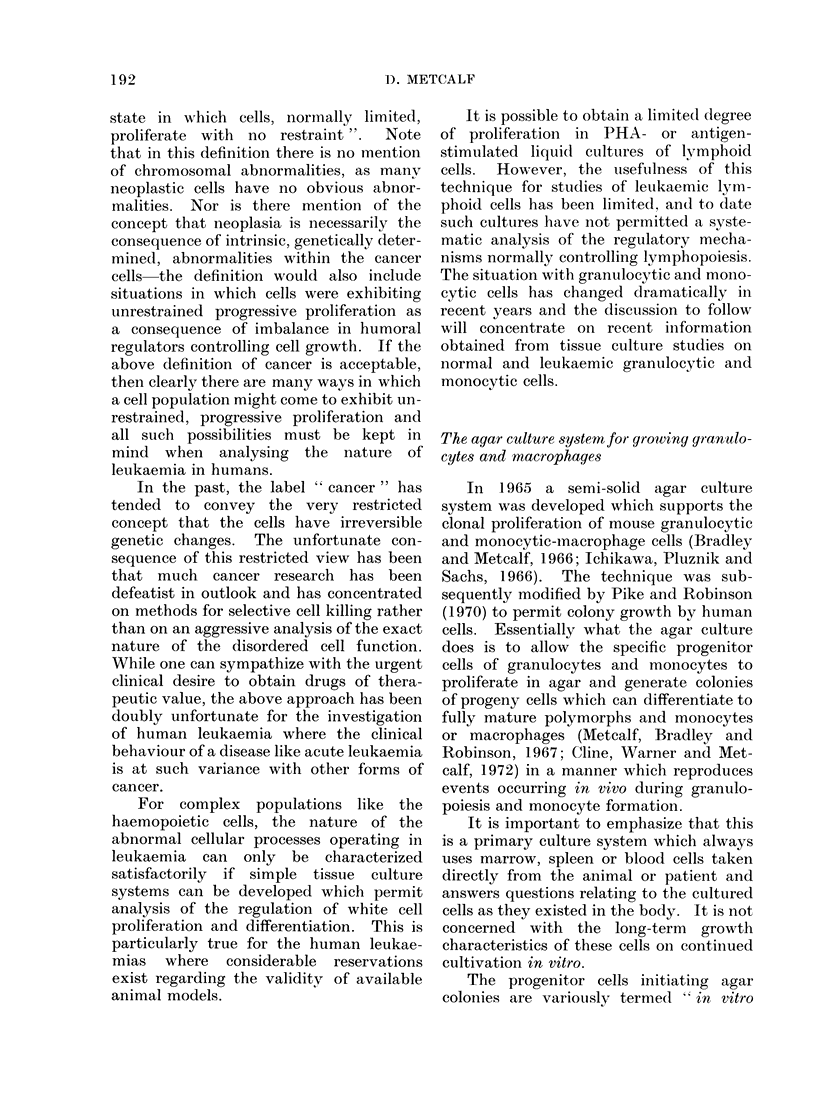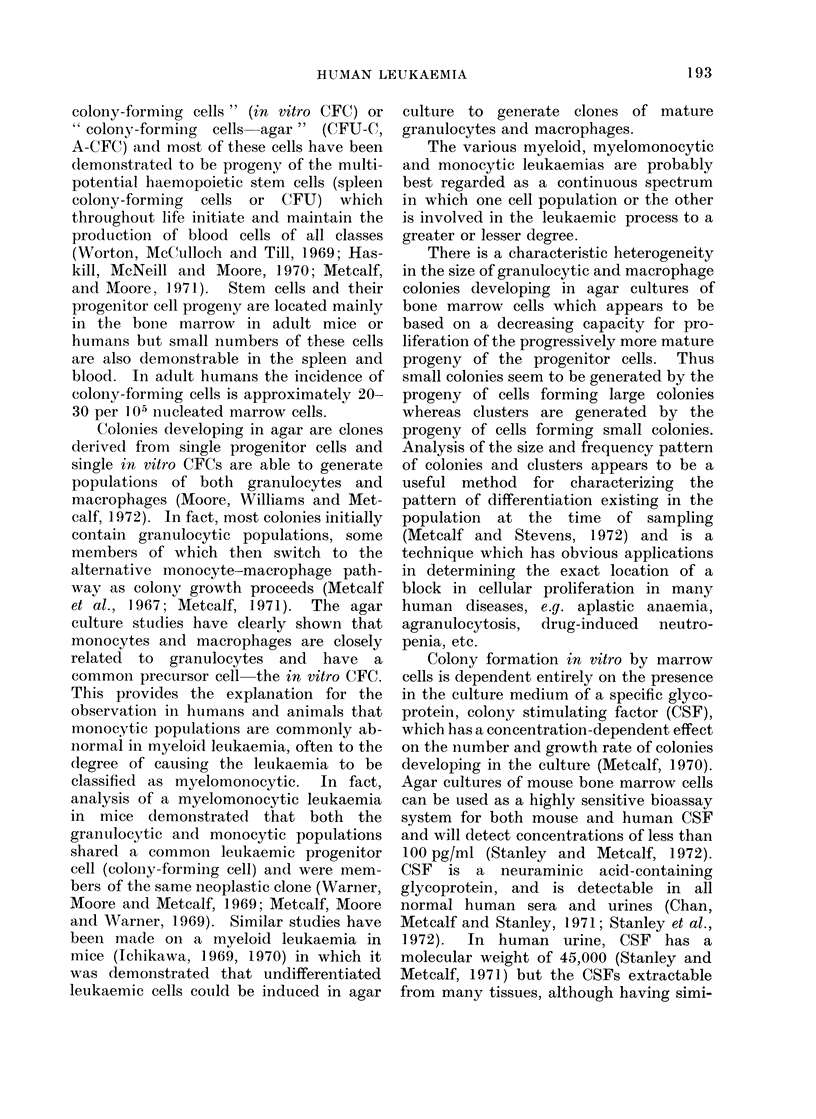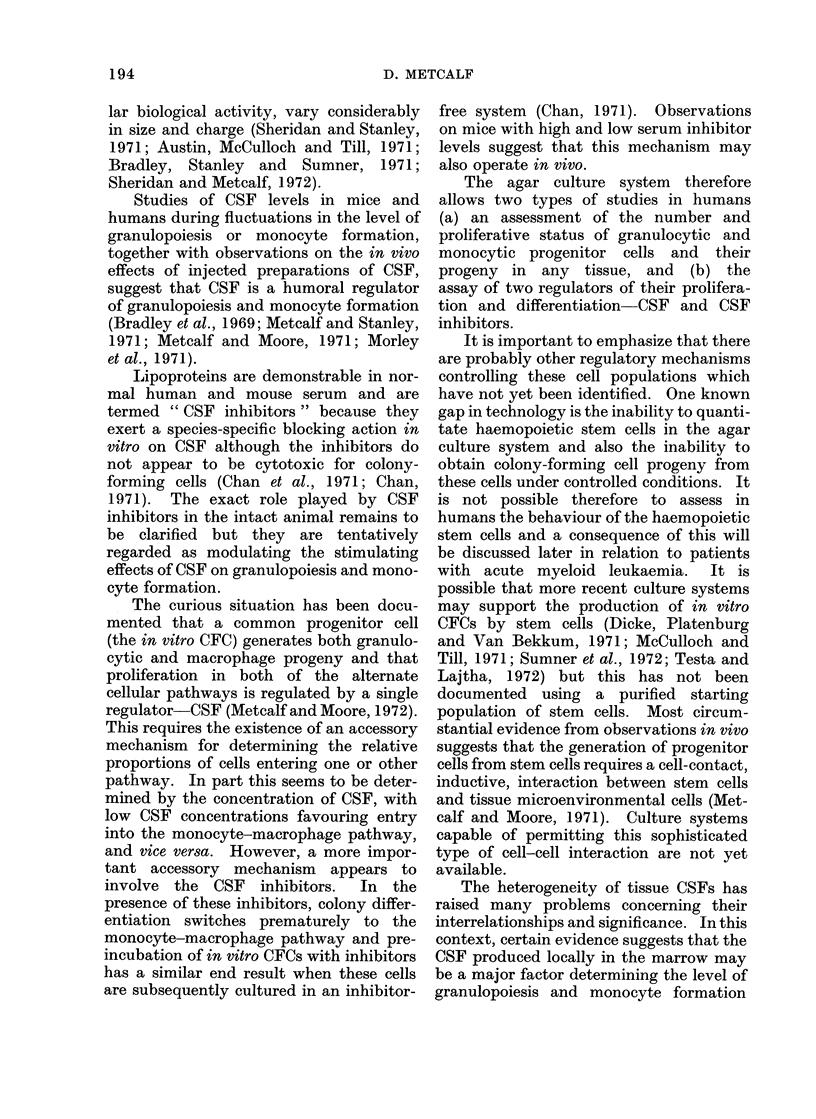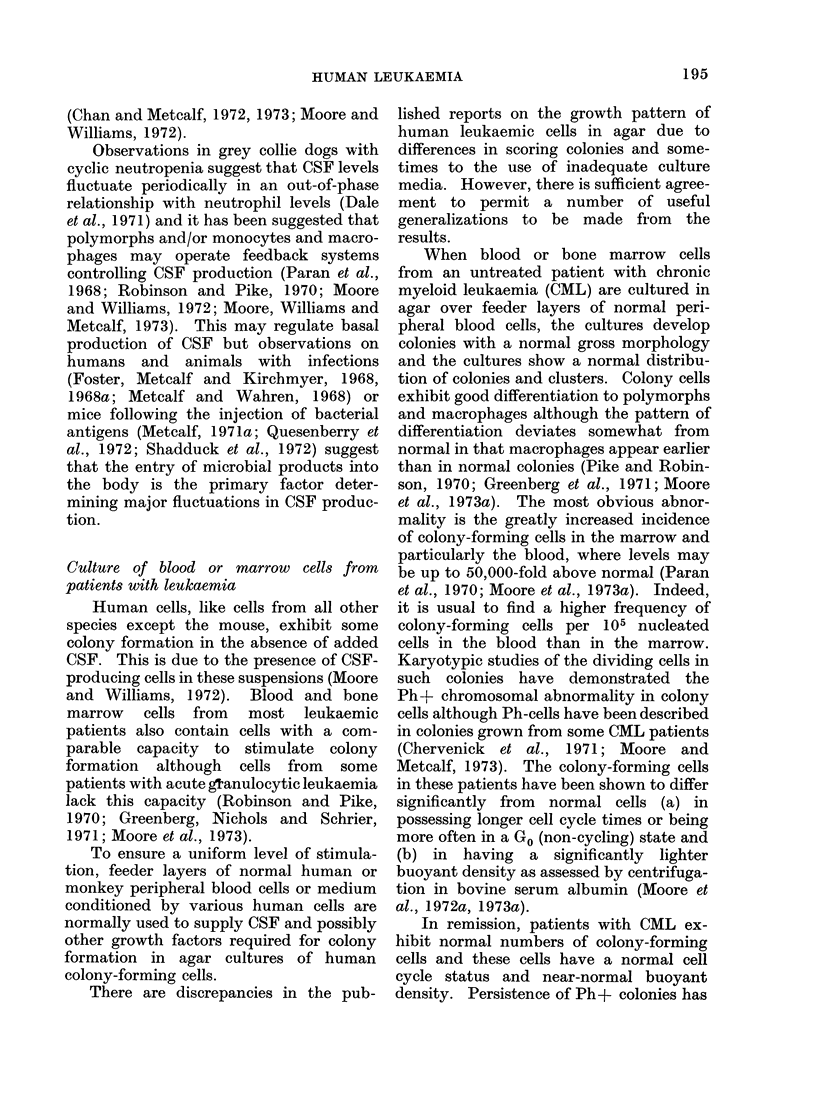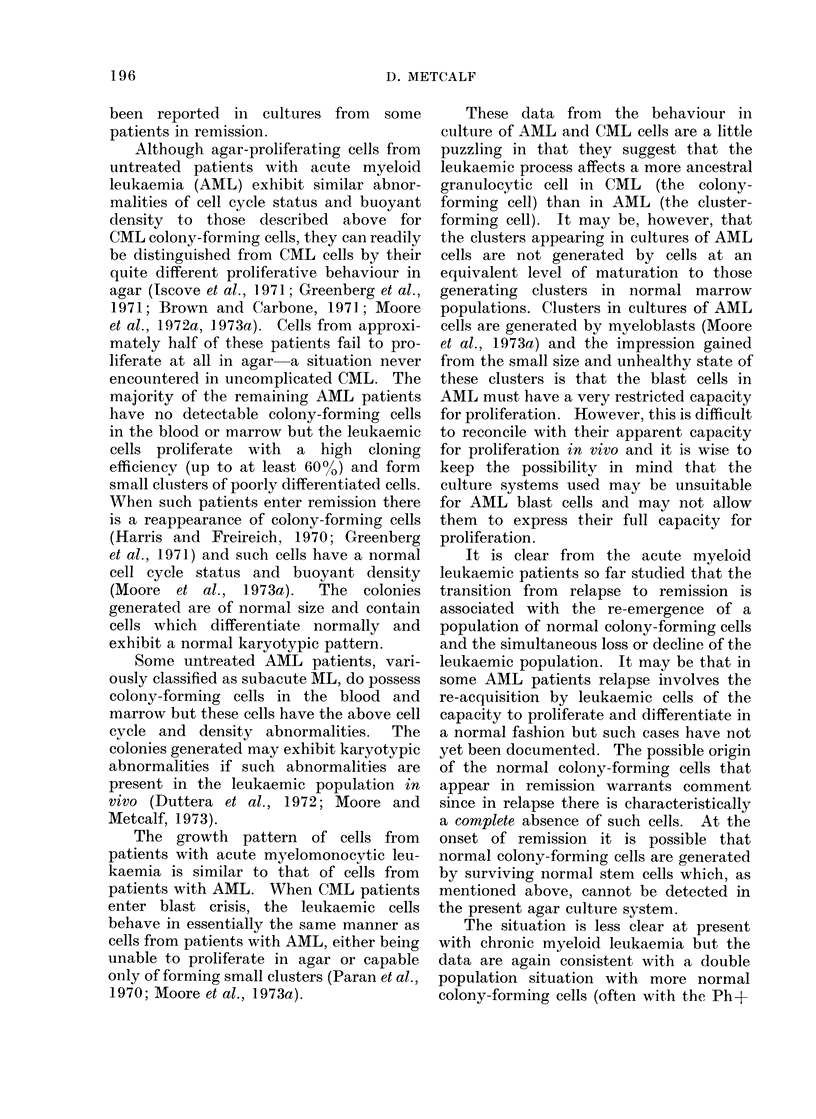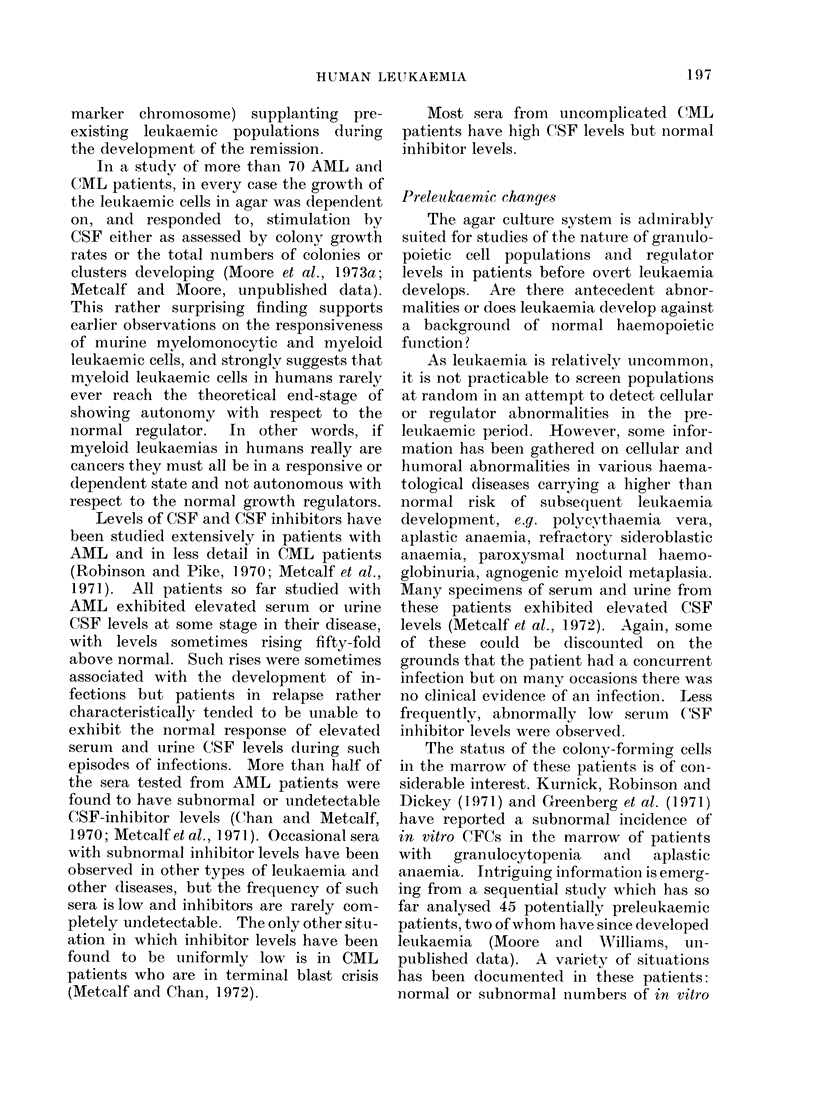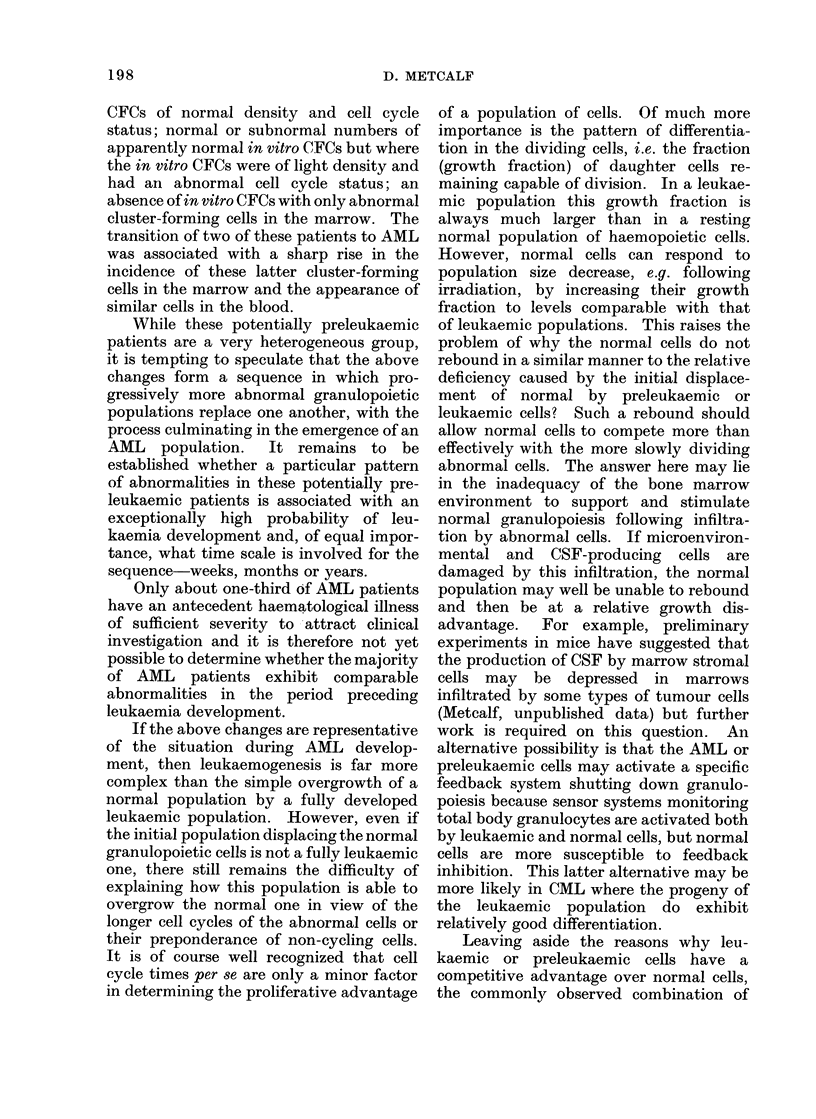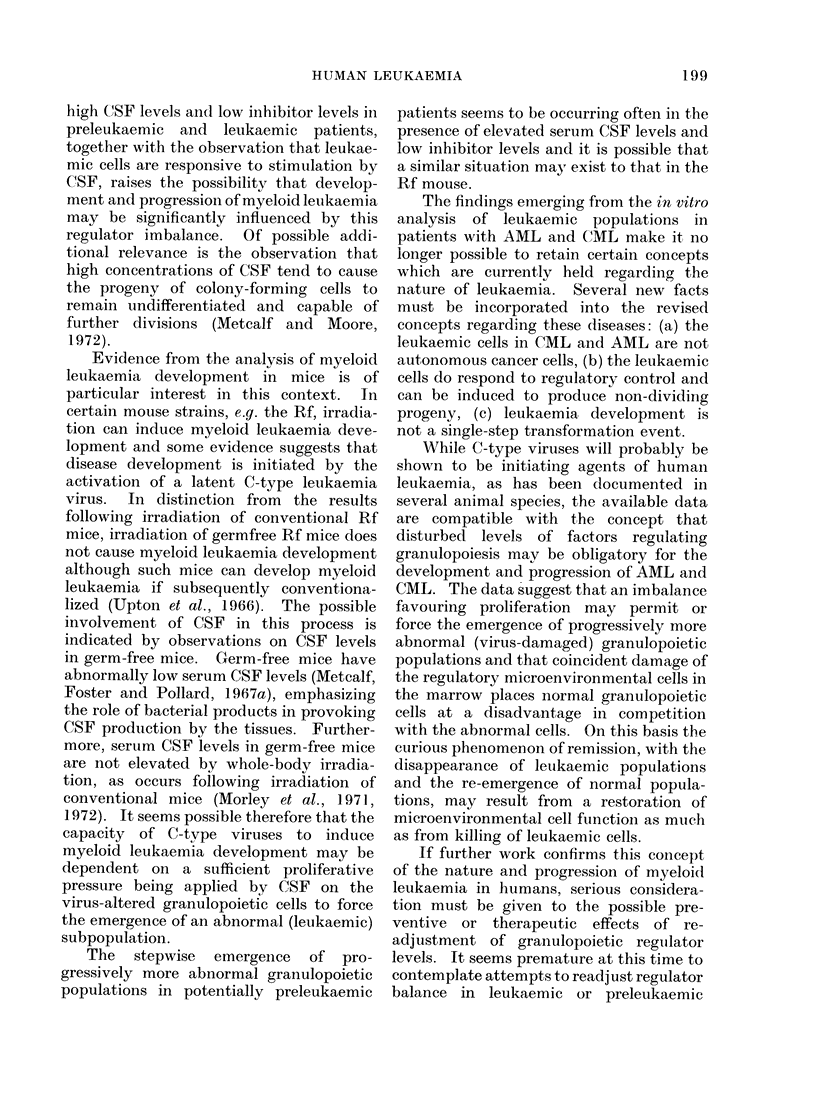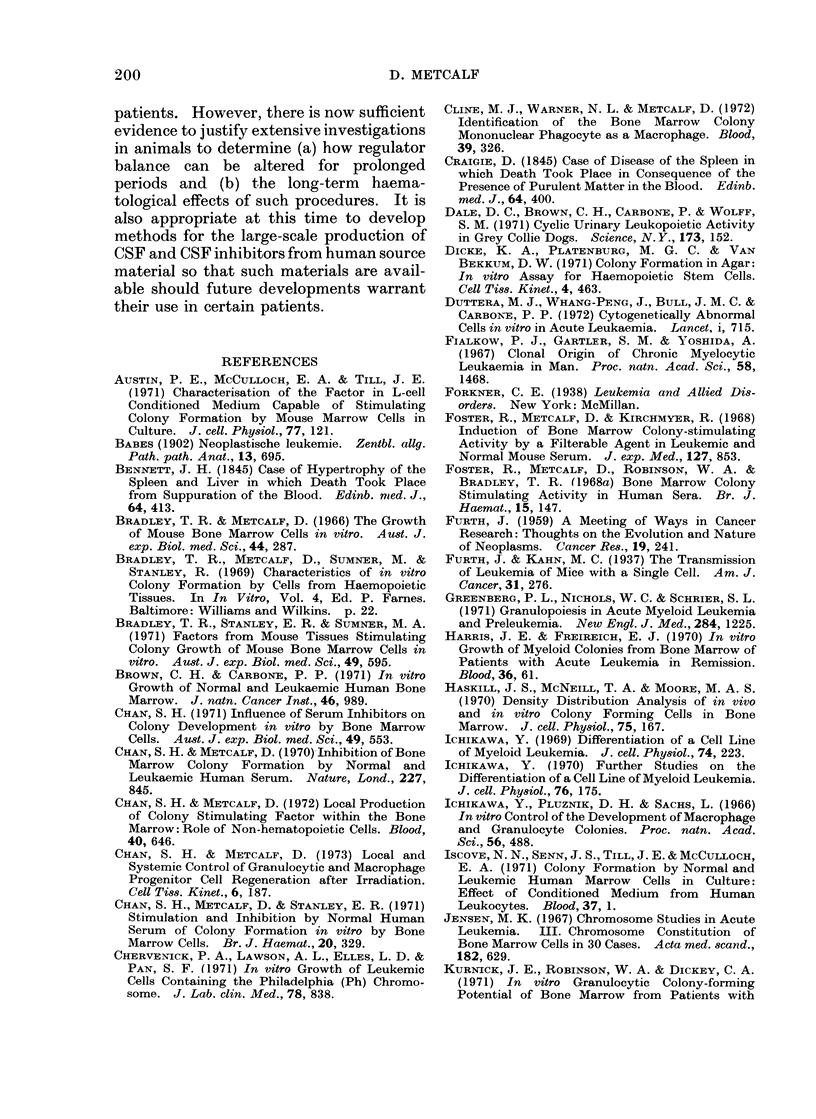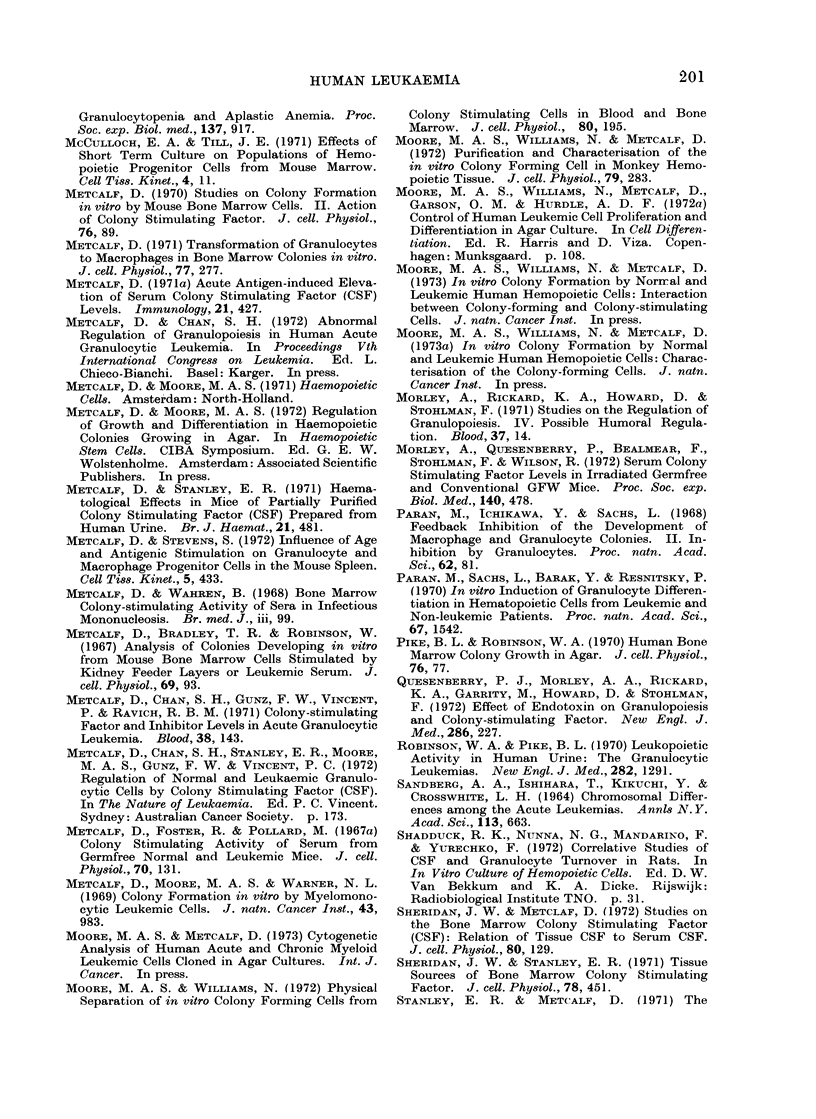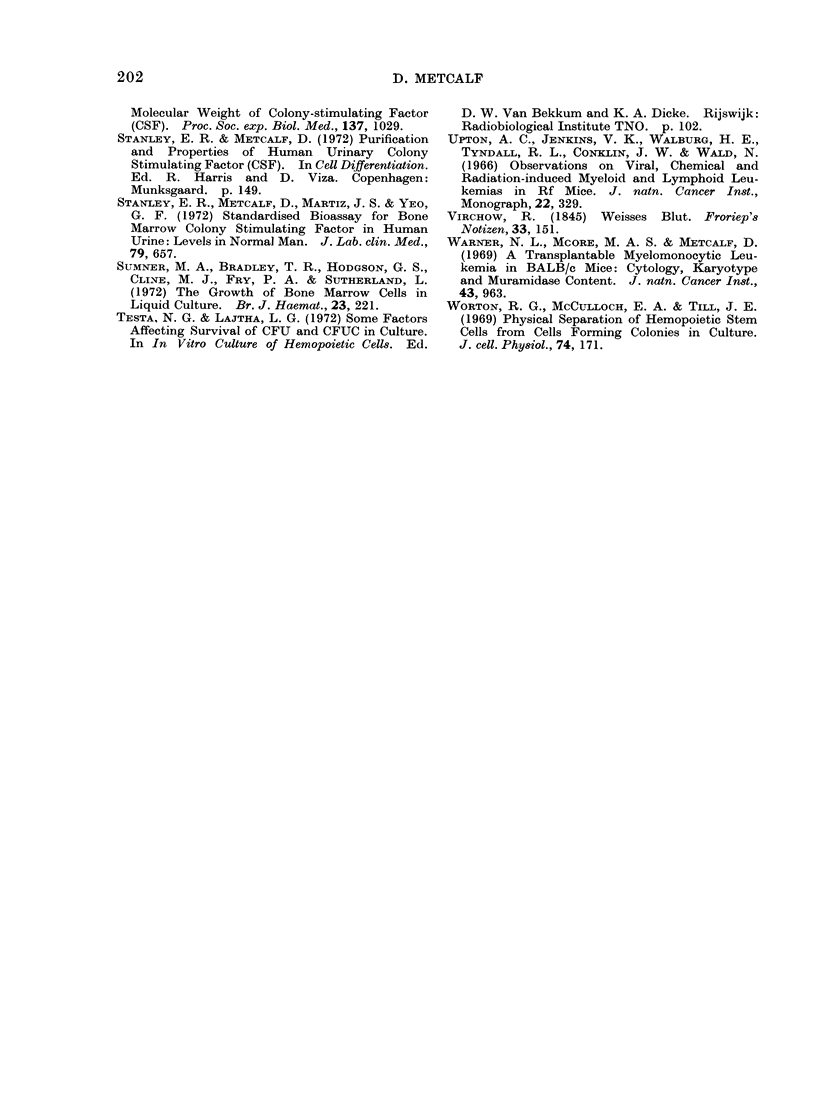# Human leukaemia: recent tissue culture studies on the nature of myeloid leukaemia.

**DOI:** 10.1038/bjc.1973.24

**Published:** 1973-03

**Authors:** D. Metcalf


Br. J. Cancer (1973) 27, 191

*Review Article*

### HUMAN LEUKAEMIA: RECENT TISSUE CULTURE STUDIES ON THE NATURE OF MYELOID LEUKAEMIA*

D. METCALF

*From the Cancer Research Unit, Walter and Eliza Hall Institute, Melbourne, Australia*

Received 27 November 1972. Accepted 28 November 1972

THE earliest recognition of leukaemia as a distinct disease entity can be dated with some accuracy to three independent reports of cases, each published in 1845, by Craigie, Bennett and Virchow. The view that the leukaemias are neoplastic diseases apparently was first proposed by Babes in 1902 and it is of interest that even as late as 1938 Forkner, in his scholarly monograph " Leukemia and Allied Disorders ", listed three major possibilities regarding the nature of leukaemia—infection, regulator disorder or neoplasm—none of which was overwhelmingly supported by the experimental or clinical evidence then available.

The view held by most workers today is that the leukaemias are neoplastic diseases. The general clinical features of the leukaemias—progressive, ultimately fatal, diseases involving the accumulation of abnormal numbers of primitive white cells, often in abnormal locations—fit the pattern of other forms of cancer. Despite the fact that none of the various forms of leukaemia in animals is a particularly accurate model of the human diseases, work on the nature of leukaemia in animals, particularly mice, has obviously had an overwhelming impact on modern views on the human diseases.

Many lines of evidence suggest that leukaemia in animals is a neoplastic disease. Agents such as irradiation and methylcholanthrene, which are able to induce carcinomata in various tissues in animals, are also leukaemogenic. Possibly the most convincing experiments proving the neoplastic nature of murine leukaemia were the demonstrations by Furth and Kahn (1937) that leukaemia could be transplanted to mice by the injection of a single leukaemic cell, and also the subsequent work of others showing that the cells of transplanted leukaemias are derived from the progressive proliferation of the original transplanted cells.

The clonal nature of an abnormal population is highly suggestive, if not conclusive, evidence for a neoplastic process. Karyotypic studies on mouse leukaemias have shown the clonal nature of some leukaemic populations and parallel studies on certain acute leukaemias in humans where the cells possessed karyotypic markers have suggested also that leukaemic populations can be clonal in nature (Sandberg *et al.*, 1964; Jensen, 1967). Recent work on patients with chronic granulocytic leukaemia who are heterozygous for the G-6-PD locus has also suggested that the leukaemic population can be clonal (Fialkow, Gartler and Yoshida, 1967). However, no information yet exists on the nature of chronic lymphoid leukaemic populations.

Attachment of the label " cancer " to a disease process is of course merely to beg the question " What is cancer? " The word appears deceptively simple until one attempts a precise definition which is sufficiently broad to cover all forms of cancer. A useful definition is still that given by Furth (1959): " Neoplasia is a

* This work was supported by the Carden Fellowship Fund of the Anti-Cancer Council of Victoria.

state in which cells, normally limited, proliferate with no restraint ". Note that in this definition there is no mention of chromosomal abnormalities, as many neoplastic cells have no obvious abnormalities. Nor is there mention of the concept that neoplasia is necessarily the consequence of intrinsic, genetically determined, abnormalities within the cancer cells—the definition would also include situations in which cells were exhibiting unrestrained progressive proliferation as a consequence of imbalance in humoral regulators controlling cell growth. If the above definition of cancer is acceptable, then clearly there are many ways in which a cell population might come to exhibit unrestrained, progressive proliferation and all such possibilities must be kept in mind when analysing the nature of leukaemia in humans.

In the past, the label " cancer " has tended to convey the very restricted concept that the cells have irreversible genetic changes. The unfortunate consequence of this restricted view has been that much cancer research has been defeatist in outlook and has concentrated on methods for selective cell killing rather than on an aggressive analysis of the exact nature of the disordered cell function. While one can sympathize with the urgent clinical desire to obtain drugs of therapeutic value, the above approach has been doubly unfortunate for the investigation of human leukaemia where the clinical behaviour of a disease like acute leukaemia is at such variance with other forms of cancer.

For complex populations like the haemopoietic cells, the nature of the abnormal cellular processes operating in leukaemia can only be characterized satisfactorily if simple tissue culture systems can be developed which permit analysis of the regulation of white cell proliferation and differentiation. This is particularly true for the human leukaemias where considerable reservations exist regarding the validity of available animal models.

It is possible to obtain a limited degree of proliferation in PHA- or antigen-stimulated liquid cultures of lymphoid cells. However, the usefulness of this technique for studies of leukaemic lymphoid cells has been limited, and to date such cultures have not permitted a systematic analysis of the regulatory mechanisms normally controlling lymphopoiesis. The situation with granulocytic and monocytic cells has changed dramatically in recent years and the discussion to follow will concentrate on recent information obtained from tissue culture studies on normal and leukaemic granulocytic and monocytic cells.

*The agar culture system for growing granulocytes and macrophages*

In 1965 a semi-solid agar culture system was developed which supports the clonal proliferation of mouse granulocytic and monocytic-macrophage cells (Bradley and Metcalf, 1966; Ichikawa, Pluznik and Sachs, 1966). The technique was subsequently modified by Pike and Robinson (1970) to permit colony growth by human cells. Essentially what the agar culture does is to allow the specific progenitor cells of granulocytes and monocytes to proliferate in agar and generate colonies of progeny cells which can differentiate to fully mature polymorphs and monocytes or macrophages (Metcalf, Bradley and Robinson, 1967; Cline, Warner and Metcalf, 1972) in a manner which reproduces events occurring *in vivo* during granulopoiesis and monocyte formation.

It is important to emphasize that this is a primary culture system which always uses marrow, spleen or blood cells taken directly from the animal or patient and answers questions relating to the cultured cells as they existed in the body. It is not concerned with the long-term growth characteristics of these cells on continued cultivation *in vitro*.

The progenitor cells initiating agar colonies are variously termed " *in vitro*

colony-forming cells " (*in vitro* CFC) or " colony-forming cells—agar " (CFU-C, A-CFC) and most of these cells have been demonstrated to be progeny of the multipotential haemopoietic stem cells (spleen colony-forming cells or CFU) which throughout life initiate and maintain the production of blood cells of all classes (Worton, McCulloch and Till, 1969; Haskill, McNeill and Moore, 1970; Metcalf, and Moore, 1971). Stem cells and their progenitor cell progeny are located mainly in the bone marrow in adult mice or humans but small numbers of these cells are also demonstrable in the spleen and blood. In adult humans the incidence of colony-forming cells is approximately 20–30 per $10^5$ nucleated marrow cells.

Colonies developing in agar are clones derived from single progenitor cells and single *in vitro* CFCs are able to generate populations of both granulocytes and macrophages (Moore, Williams and Metcalf, 1972). In fact, most colonies initially contain granulocytic populations, some members of which then switch to the alternative monocyte–macrophage pathway as colony growth proceeds (Metcalf *et al.*, 1967; Metcalf, 1971). The agar culture studies have clearly shown that monocytes and macrophages are closely related to granulocytes and have a common precursor cell—the *in vitro* CFC. This provides the explanation for the observation in humans and animals that monocytic populations are commonly abnormal in myeloid leukaemia, often to the degree of causing the leukaemia to be classified as myelomonocytic. In fact, analysis of a myelomonocytic leukaemia in mice demonstrated that both the granulocytic and monocytic populations shared a common leukaemic progenitor cell (colony-forming cell) and were members of the same neoplastic clone (Warner, Moore and Metcalf, 1969; Metcalf, Moore and Warner, 1969). Similar studies have been made on a myeloid leukaemia in mice (Ichikawa, 1969, 1970) in which it was demonstrated that undifferentiated leukaemic cells could be induced in agar culture to generate clones of mature granulocytes and macrophages.

The various myeloid, myelomonocytic and monocytic leukaemias are probably best regarded as a continuous spectrum in which one cell population or the other is involved in the leukaemic process to a greater or lesser degree.

There is a characteristic heterogeneity in the size of granulocytic and macrophage colonies developing in agar cultures of bone marrow cells which appears to be based on a decreasing capacity for proliferation of the progressively more mature progeny of the progenitor cells. Thus small colonies seem to be generated by the progeny of cells forming large colonies whereas clusters are generated by the progeny of cells forming small colonies. Analysis of the size and frequency pattern of colonies and clusters appears to be a useful method for characterizing the pattern of differentiation existing in the population at the time of sampling (Metcalf and Stevens, 1972) and is a technique which has obvious applications in determining the exact location of a block in cellular proliferation in many human diseases, *e.g.* aplastic anaemia, agranulocytosis, drug-induced neutropenia, etc.

Colony formation *in vitro* by marrow cells is dependent entirely on the presence in the culture medium of a specific glycoprotein, colony stimulating factor (CSF), which has a concentration-dependent effect on the number and growth rate of colonies developing in the culture (Metcalf, 1970). Agar cultures of mouse bone marrow cells can be used as a highly sensitive bioassay system for both mouse and human CSF and will detect concentrations of less than 100 pg/ml (Stanley and Metcalf, 1972). CSF is a neuraminic acid-containing glycoprotein, and is detectable in all normal human sera and urines (Chan, Metcalf and Stanley, 1971; Stanley *et al.*, 1972). In human urine, CSF has a molecular weight of 45,000 (Stanley and Metcalf, 1971) but the CSFs extractable from many tissues, although having similar biological activity, vary considerably in size and charge (Sheridan and Stanley, 1971; Austin, McCulloch and Till, 1971; Bradley, Stanley and Sumner, 1971; Sheridan and Metcalf, 1972).

Studies of CSF levels in mice and humans during fluctuations in the level of granulopoiesis or monocyte formation, together with observations on the *in vivo* effects of injected preparations of CSF, suggest that CSF is a humoral regulator of granulopoiesis and monocyte formation (Bradley *et al.*, 1969; Metcalf and Stanley, 1971; Metcalf and Moore, 1971; Morley *et al.*, 1971).

Lipoproteins are demonstrable in normal human and mouse serum and are termed " CSF inhibitors " because they exert a species-specific blocking action *in vitro* on CSF although the inhibitors do not appear to be cytotoxic for colony-forming cells (Chan *et al.*, 1971; Chan, 1971). The exact role played by CSF inhibitors in the intact animal remains to be clarified but they are tentatively regarded as modulating the stimulating effects of CSF on granulopoiesis and monocyte formation.

The curious situation has been documented that a common progenitor cell (the *in vitro* CFC) generates both granulocytic and macrophage progeny and that proliferation in both of the alternate cellular pathways is regulated by a single regulator—CSF (Metcalf and Moore, 1972). This requires the existence of an accessory mechanism for determining the relative proportions of cells entering one or other pathway. In part this seems to be determined by the concentration of CSF, with low CSF concentrations favouring entry into the monocyte–macrophage pathway, and *vice versa*. However, a more important accessory mechanism appears to involve the CSF inhibitors. In the presence of these inhibitors, colony differentiation switches prematurely to the monocyte–macrophage pathway and pre-incubation of *in vitro* CFCs with inhibitors has a similar end result when these cells are subsequently cultured in an inhibitor-free system (Chan, 1971). Observations on mice with high and low serum inhibitor levels suggest that this mechanism may also operate *in vivo*.

The agar culture system therefore allows two types of studies in humans (a) an assessment of the number and proliferative status of granulocytic and monocytic progenitor cells and their progeny in any tissue, and (b) the assay of two regulators of their proliferation and differentiation—CSF and CSF inhibitors.

It is important to emphasize that there are probably other regulatory mechanisms controlling these cell populations which have not yet been identified. One known gap in technology is the inability to quantitate haemopoietic stem cells in the agar culture system and also the inability to obtain colony-forming cell progeny from these cells under controlled conditions. It is not possible therefore to assess in humans the behaviour of the haemopoietic stem cells and a consequence of this will be discussed later in relation to patients with acute myeloid leukaemia. It is possible that more recent culture systems may support the production of *in vitro* CFCs by stem cells (Dicke, Platenburg and Van Bekkum, 1971; McCulloch and Till, 1971; Sumner *et al.*, 1972; Testa and Lajtha, 1972) but this has not been documented using a purified starting population of stem cells. Most circumstantial evidence from observations *in vivo* suggests that the generation of progenitor cells from stem cells requires a cell-contact, inductive, interaction between stem cells and tissue microenvironmental cells (Metcalf and Moore, 1971). Culture systems capable of permitting this sophisticated type of cell–cell interaction are not yet available.

The heterogeneity of tissue CSFs has raised many problems concerning their interrelationships and significance. In this context, certain evidence suggests that the CSF produced locally in the marrow may be a major factor determining the level of granulopoiesis and monocyte formation

(Chan and Metcalf, 1972, 1973; Moore and Williams, 1972).

Observations in grey collie dogs with cyclic neutropenia suggest that CSF levels fluctuate periodically in an out-of-phase relationship with neutrophil levels (Dale *et al.*, 1971) and it has been suggested that polymorphs and/or monocytes and macrophages may operate feedback systems controlling CSF production (Paran *et al.*, 1968; Robinson and Pike, 1970; Moore and Williams, 1972; Moore, Williams and Metcalf, 1973). This may regulate basal production of CSF but observations on humans and animals with infections (Foster, Metcalf and Kirchmyer, 1968, 1968*a*; Metcalf and Wahren, 1968) or mice following the injection of bacterial antigens (Metcalf, 1971*a*; Quesenberry *et al.*, 1972; Shadduck *et al.*, 1972) suggest that the entry of microbial products into the body is the primary factor determining major fluctuations in CSF production.

*Culture of blood or marrow cells from patients with leukaemia*

Human cells, like cells from all other species except the mouse, exhibit some colony formation in the absence of added CSF. This is due to the presence of CSF-producing cells in these suspensions (Moore and Williams, 1972). Blood and bone marrow cells from most leukaemic patients also contain cells with a comparable capacity to stimulate colony formation although cells from some patients with acute granulocytic leukaemia lack this capacity (Robinson and Pike, 1970; Greenberg, Nichols and Schrier, 1971; Moore *et al.*, 1973).

To ensure a uniform level of stimulation, feeder layers of normal human or monkey peripheral blood cells or medium conditioned by various human cells are normally used to supply CSF and possibly other growth factors required for colony formation in agar cultures of human colony-forming cells.

There are discrepancies in the published reports on the growth pattern of human leukaemic cells in agar due to differences in scoring colonies and sometimes to the use of inadequate culture media. However, there is sufficient agreement to permit a number of useful generalizations to be made from the results.

When blood or bone marrow cells from an untreated patient with chronic myeloid leukaemia (CML) are cultured in agar over feeder layers of normal peripheral blood cells, the cultures develop colonies with a normal gross morphology and the cultures show a normal distribution of colonies and clusters. Colony cells exhibit good differentiation to polymorphs and macrophages although the pattern of differentiation deviates somewhat from normal in that macrophages appear earlier than in normal colonies (Pike and Robinson, 1970; Greenberg *et al.*, 1971; Moore *et al.*, 1973*a*). The most obvious abnormality is the greatly increased incidence of colony-forming cells in the marrow and particularly the blood, where levels may be up to 50,000-fold above normal (Paran *et al.*, 1970; Moore *et al.*, 1973*a*). Indeed, it is usual to find a higher frequency of colony-forming cells per $10^5$ nucleated cells in the blood than in the marrow. Karyotypic studies of the dividing cells in such colonies have demonstrated the Ph+ chromosomal abnormality in colony cells although Ph-cells have been described in colonies grown from some CML patients (Chervenick *et al.*, 1971; Moore and Metcalf, 1973). The colony-forming cells in these patients have been shown to differ significantly from normal cells (a) in possessing longer cell cycle times or being more often in a $G_0$ (non-cycling) state and (b) in having a significantly lighter buoyant density as assessed by centrifugation in bovine serum albumin (Moore *et al.*, 1972*a*, 1973*a*).

In remission, patients with CML exhibit normal numbers of colony-forming cells and these cells have a normal cell cycle status and near-normal buoyant density. Persistence of Ph+ colonies has been reported in cultures from some patients in remission.

Although agar-proliferating cells from untreated patients with acute myeloid leukaemia (AML) exhibit similar abnormalities of cell cycle status and buoyant density to those described above for CML colony-forming cells, they can readily be distinguished from CML cells by their quite different proliferative behaviour in agar (Iscove *et al.*, 1971; Greenberg *et al.*, 1971; Brown and Carbone, 1971; Moore *et al.*, 1972*a*, 1973*a*). Cells from approximately half of these patients fail to proliferate at all in agar—a situation never encountered in uncomplicated CML. The majority of the remaining AML patients have no detectable colony-forming cells in the blood or marrow but the leukaemic cells proliferate with a high cloning efficiency (up to at least 60%) and form small clusters of poorly differentiated cells. When such patients enter remission there is a reappearance of colony-forming cells (Harris and Freireich, 1970; Greenberg *et al.*, 1971) and such cells have a normal cell cycle status and buoyant density (Moore *et al.*, 1973*a*). The colonies generated are of normal size and contain cells which differentiate normally and exhibit a normal karyotypic pattern.

Some untreated AML patients, variously classified as subacute ML, do possess colony-forming cells in the blood and marrow but these cells have the above cell cycle and density abnormalities. The colonies generated may exhibit karyotypic abnormalities if such abnormalities are present in the leukaemic population *in vivo* (Duttera *et al.*, 1972; Moore and Metcalf, 1973).

The growth pattern of cells from patients with acute myelomonocytic leukaemia is similar to that of cells from patients with AML. When CML patients enter blast crisis, the leukaemic cells behave in essentially the same manner as cells from patients with AML, either being unable to proliferate in agar or capable only of forming small clusters (Paran *et al.*, 1970; Moore *et al.*, 1973*a*).

These data from the behaviour in culture of AML and CML cells are a little puzzling in that they suggest that the leukaemic process affects a more ancestral granulocytic cell in CML (the colony-forming cell) than in AML (the cluster-forming cell). It may be, however, that the clusters appearing in cultures of AML cells are not generated by cells at an equivalent level of maturation to those generating clusters in normal marrow populations. Clusters in cultures of AML cells are generated by myeloblasts (Moore *et al.*, 1973*a*) and the impression gained from the small size and unhealthy state of these clusters is that the blast cells in AML must have a very restricted capacity for proliferation. However, this is difficult to reconcile with their apparent capacity for proliferation *in vivo* and it is wise to keep the possibility in mind that the culture systems used may be unsuitable for AML blast cells and may not allow them to express their full capacity for proliferation.

It is clear from the acute myeloid leukaemic patients so far studied that the transition from relapse to remission is associated with the re-emergence of a population of normal colony-forming cells and the simultaneous loss or decline of the leukaemic population. It may be that in some AML patients relapse involves the re-acquisition by leukaemic cells of the capacity to proliferate and differentiate in a normal fashion but such cases have not yet been documented. The possible origin of the normal colony-forming cells that appear in remission warrants comment since in relapse there is characteristically a *complete* absence of such cells. At the onset of remission it is possible that normal colony-forming cells are generated by surviving normal stem cells which, as mentioned above, cannot be detected in the present agar culture system.

The situation is less clear at present with chronic myeloid leukaemia but the data are again consistent with a double population situation with more normal colony-forming cells (often with the Ph+

marker chromosome) supplanting pre-existing leukaemic populations during the development of the remission.

In a study of more than 70 AML and CML patients, in every case the growth of the leukaemic cells in agar was dependent on, and responded to, stimulation by CSF either as assessed by colony growth rates or the total numbers of colonies or clusters developing (Moore *et al.*, 1973*a*; Metcalf and Moore, unpublished data). This rather surprising finding supports earlier observations on the responsiveness of murine myelomonocytic and myeloid leukaemic cells, and strongly suggests that myeloid leukaemic cells in humans rarely ever reach the theoretical end-stage of showing autonomy with respect to the normal regulator. In other words, if myeloid leukaemias in humans really are cancers they must all be in a responsive or dependent state and not autonomous with respect to the normal growth regulators.

Levels of CSF and CSF inhibitors have been studied extensively in patients with AML and in less detail in CML patients (Robinson and Pike, 1970; Metcalf *et al.*, 1971). All patients so far studied with AML exhibited elevated serum or urine CSF levels at some stage in their disease, with levels sometimes rising fifty-fold above normal. Such rises were sometimes associated with the development of infections but patients in relapse rather characteristically tended to be unable to exhibit the normal response of elevated serum and urine CSF levels during such episodes of infections. More than half of the sera tested from AML patients were found to have subnormal or undetectable CSF-inhibitor levels (Chan and Metcalf, 1970; Metcalf *et al.*, 1971). Occasional sera with subnormal inhibitor levels have been observed in other types of leukaemia and other diseases, but the frequency of such sera is low and inhibitors are rarely completely undetectable. The only other situation in which inhibitor levels have been found to be uniformly low is in CML patients who are in terminal blast crisis (Metcalf and Chan, 1972).

Most sera from uncomplicated CML patients have high CSF levels but normal inhibitor levels.

*Preleukaemic changes*

The agar culture system is admirably suited for studies of the nature of granulopoietic cell populations and regulator levels in patients before overt leukaemia develops. Are there antecedent abnormalities or does leukaemia develop against a background of normal haemopoietic function?

As leukaemia is relatively uncommon, it is not practicable to screen populations at random in an attempt to detect cellular or regulator abnormalities in the preleukaemic period. However, some information has been gathered on cellular and humoral abnormalities in various haematological diseases carrying a higher than normal risk of subsequent leukaemia development, *e.g.* polycythaemia vera, aplastic anaemia, refractory sideroblastic anaemia, paroxysmal nocturnal haemoglobinuria, agnogenic myeloid metaplasia. Many specimens of serum and urine from these patients exhibited elevated CSF levels (Metcalf *et al.*, 1972). Again, some of these could be discounted on the grounds that the patient had a concurrent infection but on many occasions there was no clinical evidence of an infection. Less frequently, abnormally low serum CSF inhibitor levels were observed.

The status of the colony-forming cells in the marrow of these patients is of considerable interest. Kurnick, Robinson and Dickey (1971) and Greenberg *et al.* (1971) have reported a subnormal incidence of *in vitro* CFCs in the marrow of patients with granulocytopenia and aplastic anaemia. Intriguing information is emerging from a sequential study which has so far analysed 45 potentially preleukaemic patients, two of whom have since developed leukaemia (Moore and Williams, unpublished data). A variety of situations has been documented in these patients: normal or subnormal numbers of *in vitro*

CFCs of normal density and cell cycle status; normal or subnormal numbers of apparently normal *in vitro* CFCs but where the *in vitro* CFCs were of light density and had an abnormal cell cycle status; an absence of *in vitro* CFCs with only abnormal cluster-forming cells in the marrow. The transition of two of these patients to AML was associated with a sharp rise in the incidence of these latter cluster-forming cells in the marrow and the appearance of similar cells in the blood.

While these potentially preleukaemic patients are a very heterogeneous group, it is tempting to speculate that the above changes form a sequence in which progressively more abnormal granulopoietic populations replace one another, with the process culminating in the emergence of an AML population. It remains to be established whether a particular pattern of abnormalities in these potentially preleukaemic patients is associated with an exceptionally high probability of leukaemia development and, of equal importance, what time scale is involved for the sequence—weeks, months or years.

Only about one-third of AML patients have an antecedent haematological illness of sufficient severity to attract clinical investigation and it is therefore not yet possible to determine whether the majority of AML patients exhibit comparable abnormalities in the period preceding leukaemia development.

If the above changes are representative of the situation during AML development, then leukaemogenesis is far more complex than the simple overgrowth of a normal population by a fully developed leukaemic population. However, even if the initial population displacing the normal granulopoietic cells is not a fully leukaemic one, there still remains the difficulty of explaining how this population is able to overgrow the normal one in view of the longer cell cycles of the abnormal cells or their preponderance of non-cycling cells. It is of course well recognized that cell cycle times *per se* are only a minor factor in determining the proliferative advantage of a population of cells. Of much more importance is the pattern of differentiation in the dividing cells, *i.e.* the fraction (growth fraction) of daughter cells remaining capable of division. In a leukaemic population this growth fraction is always much larger than in a resting normal population of haemopoietic cells. However, normal cells can respond to population size decrease, *e.g.* following irradiation, by increasing their growth fraction to levels comparable with that of leukaemic populations. This raises the problem of why the normal cells do not rebound in a similar manner to the relative deficiency caused by the initial displacement of normal by preleukaemic or leukaemic cells? Such a rebound should allow normal cells to compete more than effectively with the more slowly dividing abnormal cells. The answer here may lie in the inadequacy of the bone marrow environment to support and stimulate normal granulopoiesis following infiltration by abnormal cells. If microenvironmental and CSF-producing cells are damaged by this infiltration, the normal population may well be unable to rebound and then be at a relative growth disadvantage. For example, preliminary experiments in mice have suggested that the production of CSF by marrow stromal cells may be depressed in marrows infiltrated by some types of tumour cells (Metcalf, unpublished data) but further work is required on this question. An alternative possibility is that the AML or preleukaemic cells may activate a specific feedback system shutting down granulopoiesis because sensor systems monitoring total body granulocytes are activated both by leukaemic and normal cells, but normal cells are more susceptible to feedback inhibition. This latter alternative may be more likely in CML where the progeny of the leukaemic population do exhibit relatively good differentiation.

Leaving aside the reasons why leukaemic or preleukaemic cells have a competitive advantage over normal cells, the commonly observed combination of high CSF levels and low inhibitor levels in preleukaemic and leukaemic patients, together with the observation that leukaemic cells are responsive to stimulation by CSF, raises the possibility that development and progression of myeloid leukaemia may be significantly influenced by this regulator imbalance. Of possible additional relevance is the observation that high concentrations of CSF tend to cause the progeny of colony-forming cells to remain undifferentiated and capable of further divisions (Metcalf and Moore, 1972).

Evidence from the analysis of myeloid leukaemia development in mice is of particular interest in this context. In certain mouse strains, *e.g.* the Rf, irradiation can induce myeloid leukaemia development and some evidence suggests that disease development is initiated by the activation of a latent C-type leukaemia virus. In distinction from the results following irradiation of conventional Rf mice, irradiation of germfree Rf mice does not cause myeloid leukaemia development although such mice can develop myeloid leukaemia if subsequently conventionalized (Upton *et al.*, 1966). The possible involvement of CSF in this process is indicated by observations on CSF levels in germ-free mice. Germ-free mice have abnormally low serum CSF levels (Metcalf, Foster and Pollard, 1967*a*), emphasizing the role of bacterial products in provoking CSF production by the tissues. Furthermore, serum CSF levels in germ-free mice are not elevated by whole-body irradiation, as occurs following irradiation of conventional mice (Morley *et al.*, 1971, 1972). It seems possible therefore that the capacity of C-type viruses to induce myeloid leukaemia development may be dependent on a sufficient proliferative pressure being applied by CSF on the virus-altered granulopoietic cells to force the emergence of an abnormal (leukaemic) subpopulation.

The stepwise emergence of progressively more abnormal granulopoietic populations in potentially preleukaemic patients seems to be occurring often in the presence of elevated serum CSF levels and low inhibitor levels and it is possible that a similar situation may exist to that in the Rf mouse.

The findings emerging from the *in vitro* analysis of leukaemic populations in patients with AML and CML make it no longer possible to retain certain concepts which are currently held regarding the nature of leukaemia. Several new facts must be incorporated into the revised concepts regarding these diseases: (a) the leukaemic cells in CML and AML are not autonomous cancer cells, (b) the leukaemic cells do respond to regulatory control and can be induced to produce non-dividing progeny, (c) leukaemia development is not a single-step transformation event.

While C-type viruses will probably be shown to be initiating agents of human leukaemia, as has been documented in several animal species, the available data are compatible with the concept that disturbed levels of factors regulating granulopoiesis may be obligatory for the development and progression of AML and CML. The data suggest that an imbalance favouring proliferation may permit or force the emergence of progressively more abnormal (virus-damaged) granulopoietic populations and that coincident damage of the regulatory microenvironmental cells in the marrow places normal granulopoietic cells at a disadvantage in competition with the abnormal cells. On this basis the curious phenomenon of remission, with the disappearance of leukaemic populations and the re-emergence of normal populations, may result from a restoration of microenvironmental cell function as much as from killing of leukaemic cells.

If further work confirms this concept of the nature and progression of myeloid leukaemia in humans, serious consideration must be given to the possible preventive or therapeutic effects of readjustment of granulopoietic regulator levels. It seems premature at this time to contemplate attempts to readjust regulator balance in leukaemic or preleukaemic patients. However, there is now sufficient evidence to justify extensive investigations in animals to determine (a) how regulator balance can be altered for prolonged periods and (b) the long-term haematological effects of such procedures. It is also appropriate at this time to develop methods for the large-scale production of CSF and CSF inhibitors from human source material so that such materials are available should future developments warrant their use in certain patients.

### REFERENCES

AUSTIN, P. E., McCULLOCH, E. A. & TILL, J. E. (1971) Characterisation of the Factor in L-cell Conditioned Medium Capable of Stimulating Colony Formation by Mouse Marrow Cells in Culture. *J. cell. Physiol.*, **77**, 121.

BABES (1902) Neoplastische leukemie. *Zentbl. allg. Path. path. Anat.*, **13**, 695.

BENNETT, J. H. (1845) Case of Hypertrophy of the Spleen and Liver in which Death Took Place from Suppuration of the Blood. *Edinb. med. J.*, **64**, 413.

BRADLEY, T. R. & METCALF, D. (1966) The Growth of Mouse Bone Marrow Cells *in vitro*. *Aust. J. exp. Biol. med. Sci.*, **44**, 287.

BRADLEY, T. R., METCALF, D., SUMNER, M. & STANLEY, R. (1969) Characteristics of *in vitro* Colony Formation by Cells from Haemopoietic Tissues. In *In Vitro*, Vol. 4, Ed. P. Farnes. Baltimore: Williams and Wilkins. p. 22.

BRADLEY, T. R., STANLEY, E. R. & SUMNER, M. A. (1971) Factors from Mouse Tissues Stimulating Colony Growth of Mouse Bone Marrow Cells *in vitro*. *Aust. J. exp. Biol. med. Sci.*, **49**, 595.

BROWN, C. H. & CARBONE, P. P. (1971) *In vitro* Growth of Normal and Leukaemic Human Bone Marrow. *J. natn. Cancer Inst.*, **46**, 989.

CHAN, S. H. (1971) Influence of Serum Inhibitors on Colony Development *in vitro* by Bone Marrow Cells. *Aust. J. exp. Biol. med. Sci.*, **49**, 553.

CHAN, S. H. & METCALF, D. (1970) Inhibition of Bone Marrow Colony Formation by Normal and Leukaemic Human Serum. *Nature, Lond.*, **227**, 845.

CHAN, S. H. & METCALF, D. (1972) Local Production of Colony Stimulating Factor within the Bone Marrow: Role of Non-hematopoietic Cells. *Blood*, **40**, 646.

CHAN, S. H. & METCALF, D. (1973) Local and Systemic Control of Granulocytic and Macrophage Progenitor Cell Regeneration after Irradiation. *Cell Tiss. Kinet.*, **6**, 187.

CHAN, S. H., METCALF, D. & STANLEY, E. R. (1971) Stimulation and Inhibition by Normal Human Serum of Colony Formation *in vitro* by Bone Marrow Cells. *Br. J. Haemat.*, **20**, 329.

CHERVENICK, P. A., LAWSON, A. L., ELLES, L. D. & PAN, S. F. (1971) *In vitro* Growth of Leukemic Cells Containing the Philadelphia (Ph) Chromosome. *J. Lab. clin. Med.*, **78**, 838.

CLINE, M. J., WARNER, N. L. & METCALF, D. (1972) Identification of the Bone Marrow Colony Mononuclear Phagocyte as a Macrophage. *Blood*, **39**, 326.

CRAIGIE, D. (1845) Case of Disease of the Spleen in which Death Took Place in Consequence of the Presence of Purulent Matter in the Blood. *Edinb. med. J.*, **64**, 400.

DALE, D. C., BROWN, C. H., CARBONE, P. & WOLFF, S. M. (1971) Cyclic Urinary Leukopoietic Activity in Grey Collie Dogs. *Science, N.Y.*, **173**, 152.

DICKE, K. A., PLATENBURG, M. G. C. & VAN BEKKUM, D. W. (1971) Colony Formation in Agar: *In vitro* Assay for Haemopoietic Stem Cells. *Cell Tiss. Kinet.*, **4**, 463.

DUTTERA, M. J., WHANG-PENG, J., BULL, J. M. C. & CARBONE, P. P. (1972) Cytogenetically Abnormal Cells *in vitro* in Acute Leukaemia. *Lancet*, i, 715.

FIALKOW, P. J., GARTLER, S. M. & YOSHIDA, A. (1967) Clonal Origin of Chronic Myelocytic Leukaemia in Man. *Proc. natn. Acad. Sci.*, **58**, 1468.

FORKNER, C. E. (1938) *Leukemia and Allied Disorders*. New York: McMillan.

FOSTER, R., METCALF, D. & KIRCHMYER, R. (1968) Induction of Bone Marrow Colony-stimulating Activity by a Filterable Agent in Leukemic and Normal Mouse Serum. *J. exp. Med.*, **127**, 853.

FOSTER, R., METCALF, D., ROBINSON, W. A. & BRADLEY, T. R. (1968*a*) Bone Marrow Colony Stimulating Activity in Human Sera. *Br. J. Haemat.*, **15**, 147.

FURTH, J. (1959) A Meeting of Ways in Cancer Research: Thoughts on the Evolution and Nature of Neoplasms. *Cancer Res.*, **19**, 241.

FURTH, J. & KAHN, M. C. (1937) The Transmission of Leukemia of Mice with a Single Cell. *Am. J. Cancer*, **31**, 276.

GREENBERG, P. L., NICHOLS, W. C. & SCHRIER, S. L. (1971) Granulopoiesis in Acute Myeloid Leukemia and Preleukemia. *New Engl. J. Med.*, **284**, 1225.

HARRIS, J. E. & FREIREICH, E. J. (1970) *In vitro* Growth of Myeloid Colonies from Bone Marrow of Patients with Acute Leukemia in Remission. *Blood*, **36**, 61.

HASKILL, J. S., McNEILL, T. A. & MOORE, M. A. S. (1970) Density Distribution Analysis of *in vivo* and *in vitro* Colony Forming Cells in Bone Marrow. *J. cell. Physiol.*, **75**, 167.

ICHIKAWA, Y. (1969) Differentiation of a Cell Line of Myeloid Leukemia. *J. cell. Physiol.*, **74**, 223.

ICHIKAWA, Y. (1970) Further Studies on the Differentiation of a Cell Line of Myeloid Leukemia. *J. cell. Physiol.*, **76**, 175.

ICHIKAWA, Y., PLUZNIK, D. H. & SACHS, L. (1966) *In vitro* Control of the Development of Macrophage and Granulocyte Colonies. *Proc. natn. Acad. Sci.*, **56**, 488.

ISCOVE, N. N., SENN, J. S., TILL, J. E. & McCULLOCH, E. A. (1971) Colony Formation by Normal and Leukemic Human Marrow Cells in Culture: Effect of Conditioned Medium from Human Leukocytes. *Blood*, **37**, 1.

JENSEN, M. K. (1967) Chromosome Studies in Acute Leukemia. III. Chromosome Constitution of Bone Marrow Cells in 30 Cases. *Acta med. scand.*, **182**, 629.

KURNICK, J. E., ROBINSON, W. A. & DICKEY, C. A. (1971) *In vitro* Granulocytic Colony-forming Potential of Bone Marrow from Patients with

Granulocytopenia and Aplastic Anemia. *Proc. Soc. exp. Biol. med.*, **137**, 917.

McCulloch, E. A. & Till, J. E. (1971) Effects of Short Term Culture on Populations of Hemopoietic Progenitor Cells from Mouse Marrow. *Cell Tiss. Kinet.*, **4**, 11.

Metcalf, D. (1970) Studies on Colony Formation *in vitro* by Mouse Bone Marrow Cells. II. Action of Colony Stimulating Factor. *J. cell. Physiol.*, **76**, 89.

Metcalf, D. (1971) Transformation of Granulocytes to Macrophages in Bone Marrow Colonies *in vitro*. *J. cell. Physiol.*, **77**, 277.

Metcalf, D. (1971*a*) Acute Antigen-induced Elevation of Serum Colony Stimulating Factor (CSF) Levels. *Immunology*, **21**, 427.

Metcalf, D. & Chan, S. H. (1972) Abnormal Regulation of Granulopoiesis in Human Acute Granulocytic Leukemia. In *Proceedings Vth International Congress on Leukemia*. Ed. L. Chieco-Bianchi. Basel: Karger. In press.

Metcalf, D. & Moore, M. A. S. (1971) *Haemopoietic Cells*. Amsterdam: North-Holland.

Metcalf, D. & Moore, M. A. S. (1972) Regulation of Growth and Differentiation in Haemopoietic Colonies Growing in Agar. In *Haemopoietic Stem Cells*. CIBA Symposium. Ed. G. E. W. Wolstenholme. Amsterdam: Associated Scientific Publishers. In press.

Metcalf, D. & Stanley, E. R. (1971) Haematological Effects in Mice of Partially Purified Colony Stimulating Factor (CSF) Prepared from Human Urine. *Br. J. Haemat.*, **21**, 481.

Metcalf, D. & Stevens, S. (1972) Influence of Age and Antigenic Stimulation on Granulocyte and Macrophage Progenitor Cells in the Mouse Spleen. *Cell Tiss. Kinet.*, **5**, 433.

Metcalf, D. & Wahren, B. (1968) Bone Marrow Colony-stimulating Activity of Sera in Infectious Mononucleosis. *Br. med. J.*, iii, 99.

Metcalf, D., Bradley, T. R. & Robinson, W. (1967) Analysis of Colonies Developing *in vitro* from Mouse Bone Marrow Cells Stimulated by Kidney Feeder Layers or Leukemic Serum. *J. cell. Physiol.*, **69**, 93.

Metcalf, D., Chan, S. H., Gunz, F. W., Vincent, P. & Ravich, R. B. M. (1971) Colony-stimulating Factor and Inhibitor Levels in Acute Granulocytic Leukemia. *Blood*, **38**, 143.

Metcalf, D., Chan, S. H., Stanley, E. R., Moore, M. A. S., Gunz, F. W. & Vincent, P. C. (1972) Regulation of Normal and Leukaemic Granulocytic Cells by Colony Stimulating Factor (CSF). In *The Nature of Leukaemia*. Ed. P. C. Vincent. Sydney: Australian Cancer Society. p. 173.

Metcalf, D., Foster, R. & Pollard, M. (1967*a*) Colony Stimulating Activity of Serum from Germfree Normal and Leukemic Mice. *J. cell. Physiol.*, **70**, 131.

Metcalf, D., Moore, M. A. S. & Warner, N. L. (1969) Colony Formation *in vitro* by Myelomonocytic Leukemic Cells. *J. natn. Cancer Inst.*, **43**, 983.

Moore, M. A. S. & Metcalf, D. (1973) Cytogenetic Analysis of Human Acute and Chronic Myeloid Leukemic Cells Cloned in Agar Cultures. *Int. J. Cancer*. In press.

Moore, M. A. S. & Williams, N. (1972) Physical Separation of *in vitro* Colony Forming Cells from Colony Stimulating Cells in Blood and Bone Marrow. *J. cell. Physiol.*, **80**, 195.

Moore, M. A. S., Williams, N. & Metcalf, D. (1972) Purification and Characterisation of the *in vitro* Colony Forming Cell in Monkey Hemopoietic Tissue. *J. cell. Physiol.*, **79**, 283.

Moore, M. A. S., Williams, N., Metcalf, D., Garson, O. M. & Hurdle, A. D. F. (1972*a*) Control of Human Leukemic Cell Proliferation and Differentiation in Agar Culture. In *Cell Differentiation*. Ed. R. Harris and D. Viza. Copenhagen: Munksgaard. p. 108.

Moore, M. A. S., Williams, N. & Metcalf, D. (1973) *In vitro* Colony Formation by Normal and Leukemic Human Hemopoietic Cells: Interaction between Colony-forming and Colony-stimulating Cells. *J. natn. Cancer Inst.* In press.

Moore, M. A. S., Williams, N. & Metcalf, D. (1973*a*) *In vitro* Colony Formation by Normal and Leukemic Human Hemopoietic Cells: Characterisation of the Colony-forming Cells. *J. natn. Cancer Inst.* In press.

Morley, A., Rickard, K. A., Howard, D. & Stohlman, F. (1971) Studies on the Regulation of Granulopoiesis. IV. Possible Humoral Regulation. *Blood*, **37**, 14.

Morley, A., Quesenberry, P., Bealmear, F., Stohlman, F. & Wilson, R. (1972) Serum Colony Stimulating Factor Levels in Irradiated Germfree and Conventional GFW Mice. *Proc. Soc. exp. Biol. Med.*, **140**, 478.

Paran, M., Ichikawa, Y. & Sachs, L. (1968) Feedback Inhibition of the Development of Macrophage and Granulocyte Colonies. II. Inhibition by Granulocytes. *Proc. natn. Acad. Sci.*, **62**, 81.

Paran, M., Sachs, L., Barak, Y. & Resnitsky, P. (1970) *In vitro* Induction of Granulocyte Differentiation in Hematopoietic Cells from Leukemic and Non-leukemic Patients. *Proc. natn. Acad. Sci.*, **67**, 1542.

Pike, B. L. & Robinson, W. A. (1970) Human Bone Marrow Colony Growth in Agar. *J. cell. Physiol.*, **76**, 77.

Quesenberry, P. J., Morley, A. A., Rickard, K. A., Garrity, M., Howard, D. & Stohlman, F. (1972) Effect of Endotoxin on Granulopoiesis and Colony-stimulating Factor. *New Engl. J. Med.*, **286**, 227.

Robinson, W. A. & Pike, B. L. (1970) Leukopoietic Activity in Human Urine: The Granulocytic Leukemias. *New Engl. J. Med.*, **282**, 1291.

Sandberg, A. A., Ishihara, T., Kikuchi, Y. & Crosswhite, L. H. (1964) Chromosomal Differences among the Acute Leukemias. *Annls N.Y. Acad. Sci.*, **113**, 663.

Shadduck, R. K., Nunna, N. G., Mandarino, F. & Yurechko, F. (1972) Correlative Studies of CSF and Granulocyte Turnover in Rats. In *In Vitro Culture of Hemopoietic Cells*. Ed. D. W. Van Bekkum and K. A. Dicke. Rijswijk: Radiobiological Institute TNO. p. 31.

Sheridan, J. W. & Metclaf, D. (1972) Studies on the Bone Marrow Colony Stimulating Factor (CSF): Relation of Tissue CSF to Serum CSF. *J. cell. Physiol.*, **80**, 129.

Sheridan, J. W. & Stanley, E. R. (1971) Tissue Sources of Bone Marrow Colony Stimulating Factor. *J. cell. Physiol.*, **78**, 451.

Stanley, E. R. & Metcalf, D. (1971) The

Molecular Weight of Colony-stimulating Factor (CSF). *Proc. Soc. exp. Biol. Med.*, **137**, 1029.

STANLEY, E. R. & METCALF, D. (1972) Purification and Properties of Human Urinary Colony Stimulating Factor (CSF). In *Cell Differentiation*. Ed. R. Harris and D. Viza. Copenhagen: Munksgaard. p. 149.

STANLEY, E. R., METCALF, D., MARTIZ, J. S. & YEO, G. F. (1972) Standardised Bioassay for Bone Marrow Colony Stimulating Factor in Human Urine: Levels in Normal Man. *J. Lab. clin. Med.*, **79**, 657.

SUMNER, M. A., BRADLEY, T. R., HODGSON, G. S., CLINE, M. J., FRY, P. A. & SUTHERLAND, L. (1972) The Growth of Bone Marrow Cells in Liquid Culture. *Br. J. Haemat.*, **23**, 221.

TESTA, N. G. & LAJTHA, L. G. (1972) Some Factors Affecting Survival of CFU and CFUC in Culture. In *In Vitro Culture of Hemopoietic Cells*. Ed. D. W. Van Bekkum and K. A. Dicke. Rijswijk: Radiobiological Institute TNO. p. 102.

UPTON, A. C., JENKINS, V. K., WALBURG, H. E., TYNDALL, R. L., CONKLIN, J. W. & WALD, N. (1966) Observations on Viral, Chemical and Radiation-induced Myeloid and Lymphoid Leukemias in Rf Mice. *J. natn. Cancer Inst.*, Monograph, **22**, 329.

VIRCHOW, R. (1845) Weisses Blut. *Froriep's Notizen*, **33**, 151.

WARNER, N. L., MOORE, M. A. S. & METCALF, D. (1969) A Transplantable Myelomonocytic Leukemia in BALB/c Mice: Cytology, Karyotype and Muramidase Content. *J. natn. Cancer Inst.*, **43**, 963.

WORTON, R. G., MCCULLOCH, E. A. & TILL, J. E. (1969) Physical Separation of Hemopoietic Stem Cells from Cells Forming Colonies in Culture. *J. cell. Physiol.*, **74**, 171.